# Virtual mentalizing imagery therapy for Spanish language Latino family dementia caregivers: A feasibility and acceptability study

**DOI:** 10.3389/fpsyg.2023.961835

**Published:** 2023-02-16

**Authors:** Liliana Ramirez-Gomez, Julene K. Johnson, Christine Ritchie, Ashley K. Meyer, Emily Tan, Saira Madarasmi, Paulina Gutierrez-Ramirez, Cecilianna Aldarondo-Hernández, David Mischoulon, Sreya Banerjee, Felipe A. Jain

**Affiliations:** ^1^Department of Neurology, Massachusetts General Hospital, Boston, MA, United States; ^2^Department of Neurology, Harvard Medical School, Boston, MA, United States; ^3^Mongan Institute Center for Aging and Serious Illness, Massachusetts General Hospital, Harvard Medical School, Boston, MA, United States; ^4^Center for Aging in Diverse Communities, University of California, San Francisco, CA, United States; ^5^Depression Clinical and Research Program, Department of Psychiatry, Massachusetts General Hospital, Boston, MA, United States; ^6^Department of Psychiatry, Harvard Medical School, Boston, MA, United States

**Keywords:** dementia, caregiver, depression, well-being, virtual, mindfulness, mentalization, guided imagery

## Abstract

Spanish speaking family caregivers of people living with dementia have limited supportive resources in Spanish. There are few validated, culturally acceptable virtual interventions for reducing these caregivers’ psychological distress. We investigated the feasibility of a Spanish language adaptation of a virtual Mentalizing Imagery Therapy (MIT) program, which provides guided imagery and mindfulness training to reduce depression, increase mentalizing, and promote well-being. 12 Spanish-speaking family dementia caregivers received a 4-week virtual MIT program. Follow-up was obtained post group and at 4 months post baseline assessment. Feasibility, acceptability, and satisfaction with MIT were assessed. The primary psychological outcome was depressive symptoms; secondary outcomes included caregiver burden, dispositional mindfulness, perceived stress, well-being, interpersonal support, and neurological quality of life. Statistical analysis was performed with mixed linear models. Caregivers were 52 ± 8 (mean ± SD) years of age. 60% had a high school education or less. Participation in weekly group meetings was 100%. Home practice was performed on average 4 ± 1 times per week [range 2–5]. Satisfaction with MIT reached 19 ± 2 of a possible 20 points. Reduction in depression from baseline was observed by week three (*p* = 0.01) and maintained at 4 month follow-up (*p* = 0.05). There were significant improvements in mindfulness post-group, and in caregiver burden and well-being at 4 months. MIT was successfully adapted for Latino Spanish language family dementia caregivers within a virtual group environment. MIT is feasible and acceptable and may help reduce depressive symptoms and improve subjective well-being. Larger, randomized controlled trials of MIT should determine durability of effects and validate efficacy in this population.

## Introduction

In the United States, Latinos are at 1.5 times higher risk of Alzheimer’s disease (AD) and Alzheimer’s disease related disorders (ADRD) compared to non-Hispanic whites and will comprise 20% of all AD/ADRD by 2050 (Alzheimer’s Association, 2021). AD/ADRD family caregivers exhibit high levels of stress and depression (Sörensen and Conwell, 2011; Haley et al., 2020), and have a threefold increased risk of suicidal thoughts relative to non-caregivers (Czeisler et al., 2021). Latino family AD/ADRD caregivers whose primary language is Spanish (abbreviated as SLDCs, Spanish language dementia caregivers), have less access to supportive therapies and psychoeducational resources, and fewer validated options for reducing psychological distress (Ramirez Gomez et al., 2017).

“Mentalization” is a psychological construct that refers to the ability to understand oneself and others, including the links between internal mental states and observable behaviors (Fonagy et al., 2002). “Mentalizing” is the active verb form of mentalization and refers to the imaginative process of seeking to understand why people relate to and interact with others as they do (Allen et al., 2008). Mentalizing is not purely a cognitive process; much of the content of mentalizing relates to inner emotional life and how it expresses itself within complex and challenging interpersonal situations. This is to the extent that mentalizing has been referred to as holding the “heart and mind” of another in one’s own heart and mind (Fonagy, n.d.).

Caregiving for a relative with AD/ADRD poses a difficult challenge to mentalizing (Jain and Fonagy, 2020). Due to the accumulation of AD/ADRD related brain pathology, persons living with dementia exhibit declines in memory, personality, and behavior. Challenging neuropsychiatric symptoms often include irritability, anger, suspicion and paranoia, and apathy, among others. Family caregivers must reinterpret the meaning of the outward expression of these symptoms, which are often more extreme manifestations of common behaviors, in light of the AD/ADRD diagnosis. Caregivers must further identify the impact of their own actions on the person with AD/ADRD, who is often more highly sensitive to ambient noises and other sounds, and less able to process linguistic information. Therapeutic approaches that help caregivers to improve mentalization might reduce caregivers’ distress (Jain et al., 2014; McEvoy et al., 2019).

Mentalization based therapies are most frequently delivered in patient-therapist dyads or group psychotherapy settings (Allen et al., 2008; Allen, 2014). In 2014, Jain and colleagues published the first feasibility study of an 8-week intervention for caregivers that incorporated basic principles of mentalization into a sequenced protocol of mindfulness and guided imagery exercises (Jain et al., 2014). This intervention, now referred to as mentalizing imagery therapy (MIT; Jain and Fonagy, 2020), was refined and shortened to improve feasibility for caregivers of persons living with dementia (Yang et al., 2021; Jain et al., 2022; Yang et al., 2022). MIT is a manualized, 4-week mindfulness and guided imagery intervention that aims to reduce negative psychological symptoms such as depression and anxiety, and promote emotion regulation and perspective taking (Jain and Fonagy, 2020). MIT includes mindfulness exercises seeking to reduce emotional arousal (e.g., gentle stretching, breath-focused meditation), and guided imagery practices that focus on self and other understanding (Jain and Fonagy, 2020).

Two recent controlled pilot studies (N = 26; N = 46) conducted with English-speaking family AD/ADRD caregivers have found large effect sizes for reduction of depressive and anxiety symptoms (Jain et al., 2021, 2022). Both of these trials also reported consistent changes in resting state brain connectivity. Participants receiving MIT demonstrated strengthening of resting-state functional connectivity between the dorsolateral prefrontal cortex (a region of the brain involved in attention and planning) and a putative emotion regulation brain network (including dorso-and ventro-medial prefrontal cortex, ventrolateral prefrontal cortex, and regions of cerebellum; Jain et al., 2022). Furthermore, improved connectivity in this network was found to be associated with reduction in depressive symptoms and improvements in mindfulness, signifying a potential mechanistic role (Jain et al., 2022).

In the current study, we sought to adapt MIT to a virtual protocol for underserved SLDCs through an iterative refinement process. Our primary goal was to assess the feasibility and acceptability of MIT for this population. Secondarily, we aimed to identify the feasibility of administering psychological measures at baseline, post-group (1 month after baseline), and 3 months after completing the group intervention (4 month follow-up). Finally, we aimed to conduct limited efficacy testing as to whether MIT would be associated with persistent improvements in psychological symptoms, hypothesizing that post-group improvements in depressive symptoms would be retained at the four-month follow up.

## Methods

### Procedures

**Figure 1:**
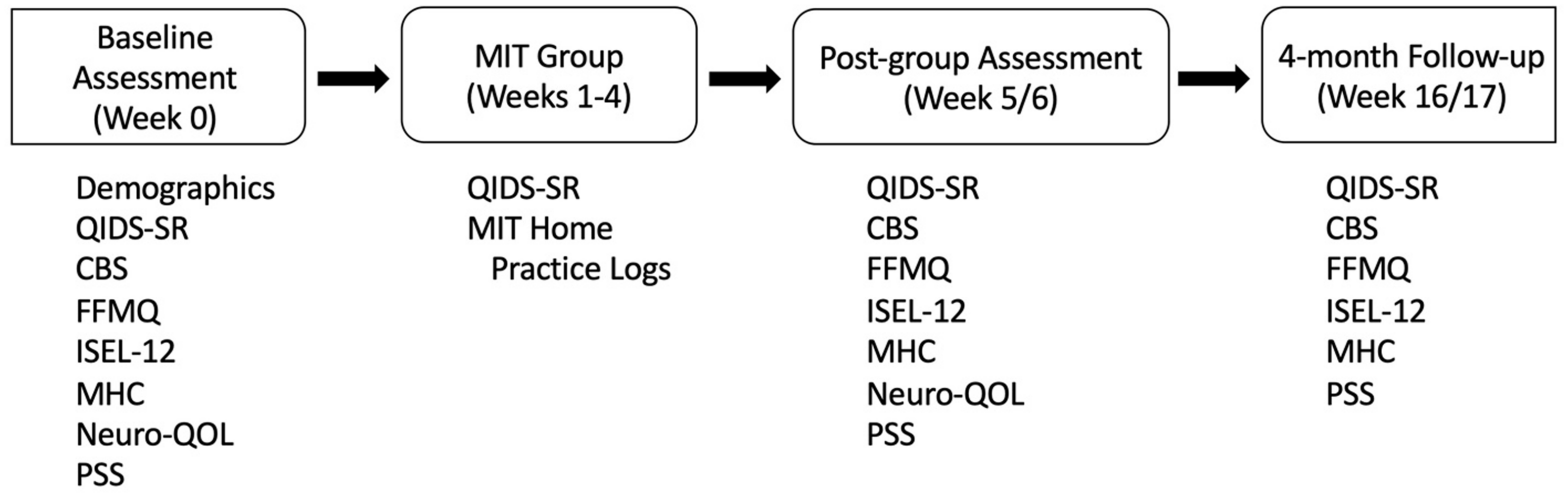
Trial flow. CBS = Caregiver Burden Scale, FFMQ = Five Facet Mindfulness Questionnaire, ISEL-12 = Interpersonal Support Evaluation List 12 item, MIT = Mentalizing Imagery Therapy, MHC = Mental Health Continuum – Short Form, Neuro-QOL = Neurological Quality of Life, PSS = Perceived Stress Scale, QIDS-SR = Quick Inventory of Depressive Symptoms – Self Report.

Team reconciliation of the MIT group manual and guided imagery instructions into Spanish was performed. Two SLDCs received the four MIT sessions in person and completed a structured debriefing interview to provide feedback on the group intervention components. Due to the COVID-19 pandemic, the materials were then further adapted for a virtual setting. We next conducted a pilot study with 12 SLDCs to test the feasibility and acceptability of the program. At baseline, within 2 weeks post group, and 4 months after baseline assessment, caregivers completed questionnaires with online REDCap software (Harris et al., 2009; Figure 1). Additionally, weekly depressive symptom and home meditation practice logs were obtained prior to the second, third and fourth group meetings using REDCap.

### Participants

All procedures were approved by the Institutional Review Board of the Massachusetts General Hospital (#2018P000798). Participants were recruited in 2019 from local clinics serving a Spanish language audience in the greater Boston area through online platforms (Facebook and Twitter), and through emailed flyers to caregiver support groups. The first group of caregivers met face-to-face in the greater Boston area. In 2020, the second stage of recruitment took place using flyers posted online. SLDCs in Medellin, Colombia, discovered the flyers and disseminated them among other known caregivers in their community. Within 3 days of posting the online flyers, more than 20 caregivers had contacted the study team. Participants were screened until the cohort was filled with the first 12 eligible participants due to funding and staffing feasibility constraints. After a detailed explanation of the study by videoconference, written informed consent was obtained electronically. Participants were screened for psychiatric disorders by a psychiatrist, and for memory impairment by a neurologist with structured assessments (please refer to online supplemental material (OSM)). Inclusion criteria included speaking Spanish as the primary language (most frequently used language), reporting elevated levels of perceived stress or reduced quality of life due to caregiving, 35 years of age or older or older, caring for a family member with dementia, and being in contact with the care recipient at least three times a week in the past year. Participants were excluded from the study if they were unable to read, had cognitive impairment, active suicidality (suicidal plan or intent to act on suicidal thoughts) within the past year, or violence toward the person living with dementia. They were also excluded if they had a current psychiatric diagnosis of schizophrenia, mania, psychotic depression, or alcohol or other substance use disorder.

Participants who were taking concomitant antidepressant or antianxiety medications were allowed to continue at current dosages. Participants were informed that they would be unable to make changes in antidepressant medication regimen, or to initiate psychotherapy or change the frequency of psychotherapy visits, between the baseline and post-group assessment visits. Participants were advised that if depressive symptoms substantially worsened during the study, they would be evaluated by a psychiatrist to determine whether they could proceed in the study or whether they would need to stop the study so as to pursue evidence-based treatments.

### Mentalizing imagery therapy intervention

For the virtual pilot, the Spanish translation of MIT was delivered by two instructors using a secure online teleconferencing platform over four consecutive weekly group meetings. Each MIT session lasted 2 hours. The primary instructor is a psychiatrist who created MIT and has more than 10 years of experience leading MIT groups. The intervention was co-led by a neurologist specialized in AD/ADRD. Audiovisual recordings of MIT exercises and the participant manual were shared on a cloud storage account for home practice. The sequence and objectives of MIT exercises have previously been described (Jain and Fonagy, 2020; Jain et al., 2022). Briefly, participants learn gentle, mindful stretching and breathing exercises that can be performed using a chair. They engage in progressively more complex meditative and guided imagery practices focused on understanding their mental processes and those of the person living with dementia, including in challenging situations. The exercises engage perspective switching and promote compassionate and empathic reflection toward the caregiver themselves, as well as the person living with dementia. Exercises also focus on recognizing how they are a part of the larger social and natural environment, including family or friends (however defined), communities (e.g., work, neighborhood), built environment, and earth. Remembrance of the participants’ own naturalistic or spiritual views on their connection with a larger source/power is encouraged.

### Measures

Pre-specified feasibility and acceptability measures included attendance at weekly group sessions, completion of home practice meditation exercises per week, a multicomponent satisfaction scale (see OSM), and semi-structured exit interviews to assess well-being and perspective-taking (Table 1). The exit interviews are the subject of a separate report. We also evaluated the feasibility of standardized clinical outcome measures. These measures included validated Spanish versions of the Quick Inventory of Depressive Symptomatology – Self Report (QIDS-SR; Gili et al., 2014), Caregiver Burden Scale (Martín-Carrasco et al., 2010), Five Factor Mindfulness Questionnaire (Cebolla et al., 2012), Mental Health Continuum – Short Form (Żemojtel-Piotrowska et al., 2018) to assess well-being, Neuro-QOL (Gershon et al., 2012) for neurological quality of life, Perceived Stress Scale (Ramírez and Hernández, 2007), and Interpersonal Support Evaluation List-12 (Merz et al., 2014) for social support.

**Table 1:** Feasibility and acceptability of MIT trial procedures and intervention.

F&A construct	Measure	Threshold	F&A threshold reached
1. Screening	# opting out; # screened by phone per week	No threshold; descriptive	N/A
2. Subject recruitment	# enrolled per week	Average 2 per week for 6 weeks	Yes
3. Subject retention	Retention rates; reasons for dropout	75% retention at final FU	Yes
4. Adherence to MIT intervention	Engagement with MIT	75% of participants will attend three in-person sessions over the first 4 weeks and will complete home practice sessions at least 2 days per week	Yes
5. Intervention fidelity	Participant satisfaction with MIT practice, degree to which MIT evoked perceived satisfaction	75% success rate for MIT practice evoking perceived satisfaction	Yes
6. Assessment protocol	Duration of battery; proportion completed; participant feedback	75% of all participants complete all assessments	Yes
7. Conditions acceptable to participants	Satisfaction survey; qualitative feedback	75% of all subjects satisfied overall; MIT rated 3+ on 5-point scale	Yes

F&A = feasibility and acceptability, FU = follow-up, N/A = not applicable, MIT = mentalizing imagery therapy.

### Statistical analysis

Data were analyzed using mixed linear models with time as a categorical fixed effect and a random factor for participant. Total scores were computed for all measures except for weekly home practice, for which an average weekly sum was obtained. Due to leftward skew of the QIDS-SR, a square root transform was applied and residuals from the resulting model examined for approximation to normality. Effect sizes from baseline to each timepoint were calculated using Cohen’s d. One participant was excluded from outcomes analyses due to the development of severe COVID-19 symptoms preventing post group assessment within the normal timeframe. Significant effects were defined as a value of *p* < 0.05.

## Results

### Phase 1. Cultural adaptation

#### Cultural adaptation of the intervention for Latino SLDCs

For the first phase of the adaptation, two SLDCs completed four in-person sessions in Spanish. Debriefing interviews were obtained at post group assessment. Participants expressed that the language and content of the materials was clear and easy to understand. Feedback from participants was positive and included statements such as, “It helped me not only caring for my relative, it also helped me when I was feeling desperate,” “It was very helpful. I need this in my life,” “It helped me sleep better. I woke up calmer. I now see things differently. I feel more peaceful,” “I loved the real examples that were provided. They felt real and I identified with them 100%.” Participants expressed satisfaction with MIT; no changes to MIT content were recommended.

### Phase 2. Feasibility and acceptability assessment

#### Demographics

All 12 SLDCs were female and monolingual Spanish speakers residing in Colombia. Caregivers were 52 ± 8 (mean ± SD) years of age [range 38–61]. For 60% of caregivers, the highest degree of educational attainment was high school completion or less. The person living with dementia was in 9 cases (75%) a parent and in 3 a grandparent, uncle, or mother-in-law. On average, caregivers reported supporting the person living with dementia for 6 ± 3 [range 1–10] years, around 86 ± 73 [range 10–168] hours per week. Four participants (33%) endorsed beginner level experience with basic meditative breathing exercises; the rest had not tried meditation practices prior to the study. No participants reported taking antidepressants or antianxiety medications. None had previously participated in support groups for dementia or utilized adult day health centers for the person living with dementia.

#### Feasibility and acceptability

All pre-specified feasibility and acceptability criteria were met (Table 1). There was 100% attendance at weekly group sessions. Caregivers remarked that the virtual format was amenable for all of them to be available at home without the need to leave their houses. Several caregivers made arrangements for other family members to be present at their homes and care for the person living with dementia while they attended the session, while others did not. Some caregivers participated in the virtual group while in the same room as the person living with dementia. Caregivers completed home practice meditation exercises on average 4 ± 1 times per week [range 2–5]. Multicomponent satisfaction with MIT achieved 19 ± 2 of a possible 20 points.

#### Depressive symptoms

Reduction in self-reported depressive symptoms from baseline was observed by the start of week 3 and maintained through post-group assessment (*d* = 0.6, *p* = 0.003, primary outcome) and 4-month follow-up (*d* = 0.5, *p* = 0.05; Table 2).

**Table 2:** Clinical outcomes.

	Timepoint	Total score	*p*	*d*
Depression	Baseline	6.1 (1.2)		
	Week 2	5.3 (0.8)	0.7	0.2
	Week 3	3 (0.5)	0.008	0.7
	Week 4	2.8 (0.45)	0.01	0.6
	Post group	2.6 (0.45)	0.003	0.6
	4 month follow up	3.6 (0.45)	0.05	0.5
Mindfulness	Baseline	142.1 (21.7)		
	Post group	154.7 (18.5)	0.02	0.6
	4 month follow up	152.7 (15.6)	0.05	0.7
Caregiver burden	Baseline	27.7 (11.7)		
	Post group	23.6 (12.5)	0.1	0.4
	4 month follow up	21.9 (11.2)	0.04	0.7
Well-being	Baseline	53.3 (12.1)		
	Post group	57.5 (6.4)	0.1	0.5
	4 month follow up	58.8 (6.6)	0.04	0.6
Perceived stress	Baseline	12.7 (5.2)		
	Post group	11.1 (3.7)	0.4	0.2
	4 month follow up	11.6 (3.4)	0.5	0.2
Interpersonal support	Baseline	34.7 (6.3)		
	Post group	37.6 (5.6)	0.1	0.7
	4 month follow up	36.7 (7.8)	0.3	0.3
Neurological quality of life	Baseline	33.7 (3.5)		
	Post group	36.4 (2.8)	0.08	0.6
	4 month follow up	Not available		

Data presented as mean (SD). *p* and *d* indicate significance and effect size of change from baseline, respectively.

#### Caregiver burden

There was a trend toward reduction in caregiver burden apparent at post group (*d* = 0.4, *p* = 0.09) with a significant improvement at 4-month follow-up (*d* = 0.7, *p* = 0.04; Table 2).

#### Mindfulness

There were significant increases in dispositional mindfulness post-group (*d* = 0.6, *p* = 0.02, Table 2). Effect size of the change was maintained at 4-month follow-up (*d* = 0.7, *p* = 0.05; Table 2).

#### Well-being

There was a trend toward improvement in well-being at post group with a moderate effect size (*d* = 0.5, *p* = 0.1). Well-being continued to improve at 4-month follow up (*d* = 0.6, *p* = 0.04; Table 2).

#### Other secondary outcomes

There were no significant improvements in perceived stress, interpersonal support, or neurological quality of life (Table 2).

## Discussion

This study provides initial evidence that MIT was successfully adapted and culturally tailored for Spanish language Latino family AD/ADRD caregivers within a virtual group environment. Evidence for feasibility was strong, with 100% caregiver attendance at weekly sessions and good compliance with home practice exercises. Moreover, multi-component satisfaction was high, suggesting acceptability in this cohort. Caregivers evidenced improvements in depressive symptoms, caregiver burden, and mindfulness. Despite a lack of ongoing group social support or additional MIT sessions after the 4-week intervention, effects were maintained at the final 4-month follow-up.

A key feature of the MIT program for SLDCs is its brevity of 4 weeks, which may allow for increased dissemination. Benefits of intervention brevity for AD/ADRD caregivers might also include increased rates of participation and adherence—which were 100% in this study. We are unaware of other brief interventions in SLDCs that have found large effect sizes for reducing depressive symptoms or improving mindfulness (Castellanos et al., 2020). A prior study of randomized 5-week cognitive-behavioral therapy versus psychoeducation for 67 SLDCs produced only “very small” effects on depression (Gonyea et al., 2016).

A second key feature of this MIT adaptation was the ability to deliver the intervention *via* a virtual platform. Due to COVID-19 containment measures, MIT had to be modified for remote delivery, and this provided an opportunity to pivot from in-person groups to virtual groups. This afforded the opportunity to expand recruitment beyond our local catchment area. To the authors’ surprise, online recruitment in the phase 2 feasibility and acceptability study resulted in recruitment of a sample of participants entirely residing in a different country (Colombia) from the research study team (United States). A recent scoping review of studies for SLDCs in Latin America showed that 90% of interventions were delivered in person (Aravena et al., 2021). Only one study used video technology during the intervention. Our results suggest that virtual delivery of therapeutic interventions to SLDCs is feasible in a South American population.

There are several limitations to this study. Although feasibility and acceptability were established within our cohort of Colombian caregivers, it is unclear how well these results might extrapolate to Latinos from other countries. Additional trials with SLDCs living in the US from a variety of countries of origin are required to provide data regarding generalizability of these findings. Because the trial was open-label and single-arm, specific efficacy of MIT components cannot be concluded. We did not assess dementia severity related factors such as activities of daily living or use of in-home supportive services which are known to impact caregiver burden. Due to the sample size, there was low power for statistical tests, resulting in likely Type II error for detection of significant effects. Although effect sizes were comparable to those of our prior research with MIT for English language caregivers (Jain et al., 2022), limitations of the sample size preclude definitive conclusions. Nevertheless, feasibility of online procedures including quantitative questionnaires was established.

In conclusion, virtual MIT was feasible and acceptable for SLDCs, with encouraging signals for reducing depressive symptoms and caregiver burden, and improving mindfulness. Despite a lack of continued group support beyond the initial 4-week training, several benefits appeared to persist at 4 months. Larger, randomized controlled trials should be performed to better characterize efficacy and durability of the intervention and to validate specific efficacy of MIT components.

## Data availability statement

The original contributions presented in the study are included in the article/Supplementary material, further inquiries can be directed to the corresponding author.

## Ethics statement

The studies involving human participants were reviewed and approved by the Institutional Review Board of the Massachusetts General Hospital (#2018P000798). The patients/participants provided their written informed consent to participate in this study.

## Author contributions

LRG designed and conducted the study, analyzed the data, drafted the manuscript, and approved the final version of the manuscript. JKJ designed the study, edited the manuscript, and approved the final version of the manuscript. CR, SM, PGR, CAH, DM, and SB edited the manuscript and approved the final version of the manuscript. AM and ET participated in data collection, edited the manuscript, and approved the final version of the manuscript. FJ designed and conducted the study, supervised all the stages of the study, analyzed the data, edited the manuscript, and approved the final version of the manuscript. All authors contributed to the article and approved the submitted version.

## Funding

LRG was supported by the National Institute on Aging pilot project from the Center for Aging in Diverse Communities at UCSF 2P30AG015272-21, the diversity supplement # 3P30AG062421-01S1, R01AG066823-01A1, 5P30AG062421-03, the Alzheimer’s Association Grant AACSFD-21-853089 and has received honoraria for speaking from the Massachusetts Neurological Association and the American Academy of Neurology. FJ was supported by the National Institute on Aging #K76AG064390, and has received honoraria for speaking from the Massachusetts General Hospital (MGH) Benson-Henry Institute for Mind/Body Medicine, and honoraria from Fondazione Cariplo for serving as a grant reviewer. He discloses work with the non-profit Clinical Trials Network Institute (CTNI) at MGH, which receives funding from pharmaceutical companies and the NIH. CR has received funding from the National Institute on Aging, the National Institute for Nursing Research, and the John A. Hartford Foundation. She has served as a consultant for the West Health Institute. DM has received research support from Nordic Naturals and heckel medizintechnik GmbH. He has received honoraria for speaking from the MGH Psychiatry Academy. He discloses work with the non-profit Clinical Trials Network. The authors declare that heckel medizintechnik GmbH was not involved in the study design, collection, analysis, interpretation of data, the writing of this article or the decision to submit it for publication.

## Conflict of interest

The authors declare that the research was conducted in the absence of any commercial or financial relationships that could be construed as a potential conflict of interest.

## Publisher’s note

All claims expressed in this article are solely those of the authors and do not necessarily represent those of their affiliated organizations, or those of the publisher, the editors and the reviewers. Any product that may be evaluated in this article, or claim that may be made by its manufacturer, is not guaranteed or endorsed by the publisher.
