# Supplementary material for: Virtual mentalizing imagery therapy for Spanish language Latino family dementia caregivers: A feasibility and acceptability study
Source: Front Psychol. 2023 Feb 16;14:961835. doi: 10.3389/fpsyg.2023.961835 (PMC9979537; doi:10.3389/fpsyg.2023.961835)
Supplement: Supplementary file 1 [file Data_Sheet_1.PDF]

Supplemental Methods and Results

Virtual mentalizing imagery therapy for Spanish language Latino family dementia caregivers: a feasibility and acceptability study

Methods:

Screening. Participants were screened for psychiatric disorders with the Spanish-language MINI International Neuropsychiatric Interview version 6.0 (1) by a psychiatrist with more than 10 years of research experience. Participants were screened for memory impairment by a neurologist with more than 10 years of research experience with a validated Spanish language translation of the Mini-Mental Status Examination (2).

Translation. We used a certified, professional English – Spanish translator to translate all MIT materials that were initially created in English. Following this translation, Dr. Liliana Ramirez Gomez, MD, who is a native Spanish speaker and also a fluent English speaker, read and reviewed in detail all the materials for accuracy. Minimal changes were required. Dr. Felipe Jain, MD, the creator of MIT, is also fully fluent in Spanish for reading, writing, and speaking, and he reviewed, further edited by consensus with Dr. Ramirez Gomez, and provided final approval to all translations of MIT materials.

Table S1. *Multicomponent satisfaction scale (administered post group)*

	Poor/ Mala	Fair/ Más o menos	Good/ Buena	Very Good/ Muy buena	Excellent/ Excelente
1. How would you rate the overall quality of the *Mentalizing Imagery Therapy*? / **En general, ¿cómo**	☐ 1	☐ 2	☐ 3	☐ 4	☐ 5

	Poor/ Mala	Fair/ Más o menos	Good/ Buena	Very Good/ Muy buena	Excellent/ Excelente
**evaluaría la calidad de la *Terapia de Mentalización con imágenes para cuidadores*?**					
2. How would you rate the overall quality of the *Mentalizing Imagery Therapy* workbook? / **En general, ¿cómo evaluaría la calidad del manual de la *Terapia de Mentalización con imágenes para cuidadores*?**	☐ 1	☐ 2	☐ 3	☐ 4	☐ 5
3. How would you rate the overall quality of the *Mentalizing Imagery Therapy* CD audio recordings? / **En general, ¿cómo evaluaría la calidad de la grabacion de audio de la *Terapia de Mentalización con imágenes para cuidadores*?**	☐ 1	☐ 2	☐ 3	☐ 4	☐ 5
4. How would you rate the skills of your *Mentalizing Imagery Therapy* instructor? / **¿Cómo evaluaría las habilidades de su instructor de *la Terapia de Mentalización con imágenes para cuidadores*?**	☐ 1	☐ 2	☐ 3	☐ 4	☐ 5

Supplemental References:

1. Sheehan D, Janavs J, Harnett-Sheehan K, et al.: M.I.N.I Entrevista Neuropsiquiátrica Internacional2010;
2. Rosselli D, Ardila A, Pradilla G, et al.: [The Mini-Mental State Examination as a selected diagnostic test for dementia: a Colombian population study. GENECO]. Rev Neurol 2000; 30:428–432
